# Colonic Volvulus in Children: Surgical Management of a Challenging Condition

**DOI:** 10.3390/children8110982

**Published:** 2021-10-30

**Authors:** Francesca Destro, Luciano Maestri, Milena Meroni, Alessandro Campari, Federica Pederiva, Sara Costanzo, Giulia Del Re, Margherita Roveri, Gianvincenzo Zuccotti, Valeria Calcaterra, Gloria Pelizzo

**Affiliations:** 1 Pediatric Surgery Department, “Vittore Buzzi” Children’s Hospital, 20154 Milano, Italy; francesca.destro@asst-fbf-sacco.it (F.D.); luciano.maestri@asst-fbf-sacco.it (L.M.); milena.meroni@asst-fbf-sacco.it (M.M.); federica.pederiva@asst-fbf-sacco.it (F.P.); sara.costanzo@asst-fbf-sacco.it (S.C.); giulia.delre@live.com (G.D.R.); margherita.roveri@studenti.unimi.it (M.R.); 2 Pediatric Radiology Department, “Vittore Buzzi” Children’s Hospital, 20154 Milano, Italy; alessandro.campari@asst-fbf-sacco.it; 3 Pediatric Department, “Vittore Buzzi” Children’s Hospital, 20154 Milano, Italy; gianvincenzo.zuccotti@unimi.it (G.Z.); valeria.calcaterra@unipv.it (V.C.); 4 Department of Biomedical and Clinical Science “L. Sacco”, University of Milano, 20157 Milano, Italy; 5 Pediatrics and Adolescentology Unit, Department of Internal Medicine, University of Pavia, 27100 Pavia, Italy

**Keywords:** colonic volvulus, children, pediatric surgery, management

## Abstract

Colonic volvulus (CV) is a rare but potentially life-threatening condition with unclear etiopathogenesis. To date, less than 80 pediatric cases have been described. Hirschsprung’s disease (HD) is associated with CV in 17% of cases, representing a significant risk factor. Non-HD CV is an even more complex entity. The aim of this study is to describe a series of patients with CV to accentuate some peculiar aspects of this disease. We performed a retrospective study (period: 2012–2021) collecting information of patients with CV. Data analyzed included: demographics, medical history, presenting symptoms and radiological and surgical details. Eleven patients (12.5 ± 2.8 years; 7F/4M) had CV (eight sigmoid, two transverse colon, one total colon). Five patients had associated anomalies and three had HD. A two-step approach with volvulus endoscopic/radiological detorsion followed by intestinal resection was attempted in eight cases (one endoscopic approach failed). Three patients required surgery at admission. At follow-up, two patients developed recurrent intestinal obstruction, one of whom also had anastomotic stenosis. Colonic volvulus is a challenging condition that requires prompt patient care. A missed diagnosis could lead to severe complications. The evaluation of the patient should include a careful histological examination (searching for HD and alpha-actin deficiency), immunologic and metabolic screening, neurological tests and detection of chronic intestinal pseudo-obstruction (CIPO). Lifelong follow-up is mandatory for the early recognition and treatment of progressive diseases involving the proximal gastrointestinal tract.

## 1. Introduction

Colonic volvulus (CV) is a potentially life-threatening condition characterized by the twisting of the large bowel on its mesenteric axis, causing an intestinal obstruction which might lead to ischemia and perforation if unrecognized [1,2,3,4]. It can involve every segment of the large bowel; however, the sigmoid colon is mostly commonly affected (60–75% of cases) [5,6]. As a disease characterized by its insidiousness, CV often goes unrecognized, resulting in delayed diagnosis and, subsequently, high morbidity and mortality [7,8]. The literature about CV in infancy and childhood is scant, and, therefore, our knowledge is largely based on adult studies and series [9,10,11,12,13].

The etiology of CV remains unclear. Several risk factors have been proposed, including differences of local anatomy (e.g., elongated mesentery with narrow attachment, excessive mobilization of colonic segments), Hirschsprung’s disease (HD), omphalomesenteric abnormalities, intestinal malrotation, anal stenosis, chronic constipation, surgical adhesions, prune belly syndrome and developmental delay [7,8,14,15,16,17,18,19]. In particular, HD is associated with CV in 17% of cases, representing a significant risk factor [7,18,19].

Management modalities and timing remain controversial. Recent series suggest non-surgical reduction (radiological and/or endoscopic) in the absence of complications (bowel necrosis, perforation) followed by elective surgery to prevent recurrences [13,20]. Data on long-term follow-up remain scarce.

In this single-center retrospective review, we analyzed our series of patients affected with CV. Preoperative risk factors are discussed to optimize the diagnosis and to avoid post operative complications. Radiological aspects were also analyzed discussing possible clinical implications. Multidisciplinary long-term follow-up is imperative to reduce future risk of recurrence.

## 2. Materials and Methods

Data was retrospectively analyzed from August 2012 to August 2021, in patients who were admitted to our Department of Pediatric Surgery (Buzzi Children’s Hospital, Milan, Italy) with a diagnosis of CV.

We considered demographic (age, gender) and clinical data (presence of associated anomalies or pathologies, symptoms at diagnosis), risk factors (previous abdominal surgery, constipation, excessive colonic mobility, HD) and diagnostic modalities in all patients.

Additionally, surgical details, time between presentation and surgery were also reviewed, together with complications, outcomes and recurrences.

The diagnosis was formulated on the basis of the clinical presentation, plain X-ray followed by contrast enema and, in complex patients, CT and/or MRI scans.

Contrast enema images were reviewed by an expert pediatric radiologist in order to define the entity and extension of colonic dilatation.

Surgery was performed as soon as possible when the contrast enema was not resolutive. In all other cases, surgery was planned after a three-month observation period. Intestinal biopsies were obtained to rule out HD.

### Statistical Analysis

Qualitative variables were described as counts and percentages. Quantitative variables were expressed as the mean value when normally distributed and compared using the Student’s *t*-test. The data analysis was performed with the STATA statistical package (release 15.1, 2017, Stata Corporation, College Station, TX, USA).

## 3. Results

### 3.1. Clinical Features

Eleven patients (12.5 ± 2.8 years; 7F/4M) came to our attention for colonic volvulus: 8/11 (72.8%) sigmoid volvulus, 2/11 (18.2%) volvulus of the transverse colon, and 1/11 (11.9%) volvulus of the entire colon.

One patient with sigmoid volvulus had simultaneous volvulus of the spleen, pancreatic tail and stomach. In five cases (5/11, 45.4%), there were associated anomalies, as described in Table 1.

Two patients underwent previous abdominal surgery (laparoscopic Nissen fundoplication and gastrostomy tube placement; ventriculoperitoneal shunt placement).

Nine patients (9/11, 82%) reported constipation according to the Rome IV criteria (at least two of the following symptoms, once per week, over the preceding month: two or fewer defecations in the toilet per week, at least one episode of fecal incontinence per week, history of retentive posturing or excessive volitional stool retention, history of painful or hard bowel movements, presence of a large fecal mass in the rectum, history of large diameter stools that may obstruct the toilet).

Upon arrival, all patients presented signs of abdominal obstruction with abdominal pain (11/11, 100%). Other frequent clinical signs included abdominal distension (10/11, 90.9%) and nausea and/or vomiting (8/11, 72.7%). Clinical examination showed an empty rectum in all patients (11/11, 100%). None of the patients had signs or symptoms of complications or perforations.

Histology for HD resulted as positive (presence of acetylcholinesterase activity) in three cases (27.3%).

### 3.2. Instrumental Results

**Figure 1:**
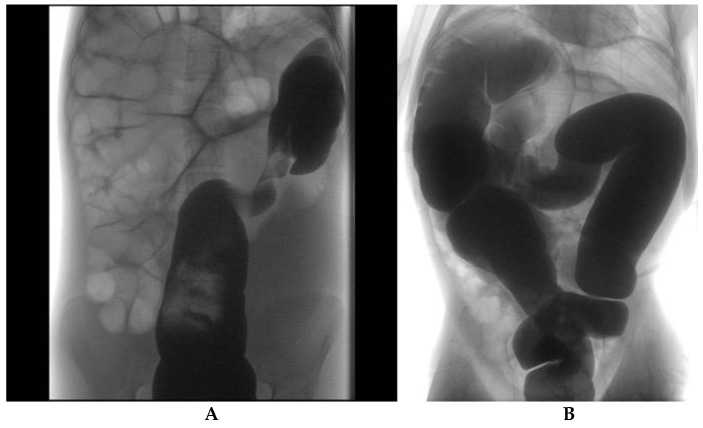
(**A**) Sigmoid colon volvulus in a 14-year-old girl. Fluoroscopic contrast enema shows visceral twisting of the distal sigmoid and gas-filled upstream bowel loops. (**B**) Transverse colon volvulus in a 13-year-old girl. Fluoroscopic contrast enema demonstrates visceral twisting of the transverse colon.

**Figure 2:**
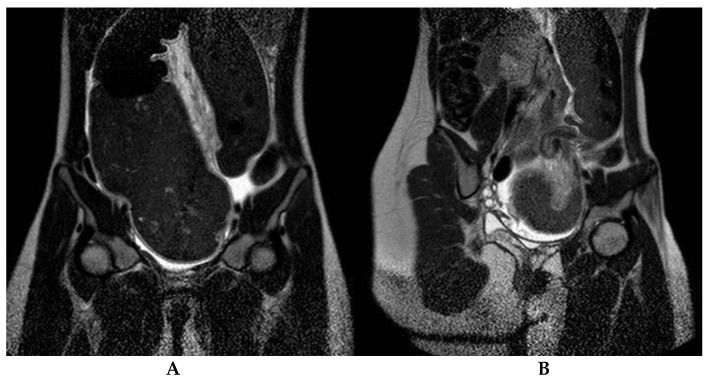
Sigmoid colon volvulus in a 14-year-old girl. Coronal T2 weighted MRI images demonstrate closed-loop obstruction of the sigmoid colon with severe visceral dilatation (**A**) and whirlpool sign (**B**); abdominal free fluid is also depicted.

Radiological evaluation included abdominal X-ray (11/11, 100%), bowel enema (10/11, 90.9%, Figure 1) and abdominal CT (2/11, 18.1%) or MRI (1/11, 9%, Figure 2) in patients with associated anomalies and an equivocal clinical picture. Typical features, namely “coffee bean” and “bird’s beak” sign, were identified in 7 and 10 cases, respectively. The mean diameter of the colonic segments is reported in Table 2. Sigmoid diameter was higher in HD patients compared to non-HD subjects (*p* = 0.02); no significant differences in other diameters were detected between the two groups.

The small intestine appeared normal in all patients.

### 3.3. Surgical Management

First-line treatment for sigmoid volvulus (eight patients) consisted of flexible sigmoidoscopy under general anesthesia in 4/8 cases (50%), radiological detorsion in 3/8 patients (37.5%) and surgical detorsion together with splenectomy and appendicostomy in 1/8 patient (12.5%) with associated volvulus of the spleen, stomach and pancreatic tail. Flexible sigmoidoscopy was successful in all but two patients (2/4 patients, 50%): one (HD positive) immediately underwent sigmoid resection and colostomy (open approach due to intestinal wall ischemic area identified during sigmoidoscopy), while the other developed recurrence of the volvulus, managed by laparoscopic left hemicolectomy. The remaining five patients (one refused surgery) had the definitive operation after a mean of 4.3 months (three sigmoid resection, one total colectomy with temporary ileostomy and one Soave pull-through). The procedures were performed laparoscopically; however, one patient with massive intestinal dilatation required conversion.

Patients with volvulus involving more proximal colon (one total colonic volvulus and two transverse colon volvulus) underwent surgical detorsion with colostomy (one case) or colopexy (two cases).

All patients were thoroughly evaluated prior to definitive surgery in order to exclude congenital anomalies, connective tissue diseases, alpha-actin deficiency, Hirschsprung’s disease and motility intestinal disorders (intestinal biopsies, cerebral MRI, genetics testing, cardiological and endocrinological evaluation).

Surgical details, together with outcomes, are reported in Table 3.

### 3.4. Follow-Up

Mean follow-up length was 5 ± 3.5 years. Patients were closely followed at our outpatient clinic (first follow-up one week after discharge and then after 1, 3 and 6 months or more often, depending on the clinical picture). Radiologists, gastroenterologists, endocrinologists, geneticists, pathologists, neurologists and neuropsychiatrists were consistently involved in multidisciplinary discussions and patient evaluations. At 11 months follow-up, one patient with autism developed anastomotic stenosis and progressive distension of the proximal ileum. She underwent redo anastomosis and multiple biopsies to further exclude CIPO disease (with no resulting evidence of CIPO or associated diseases).

Both patients with transverse colon volvulus (one of them HD positive) developed multiple episodes of intestinal obstruction after surgery and both, eventually, required total colectomy with ileostomy.

No correlation was found between HD and the development of complications.

## 4. Discussion

This is a retrospective review of our single-center experience that emphasizes how CV is to be considered a potential surgical emergency requiring prompt identification and management. In this series, all patients presented with signs and symptoms of acute intestinal obstruction and 81.8% required an urgent radiologic procedure or a surgical approach after failure of non-invasive volvulus detorsion. On radiological evaluation, different colonic diameters of the HD group were recorded when compared to patients without HD. These findings, never described before, lead us to support the view that different mechanisms could be involved in CV pathogenesis in HD compared to non-HD groups. Our results enlighten the need for an extensive evaluation of patients, including the CV functional aspects that are unclear today and deserve to be studied. A multidisciplinary approach with long-term follow-up, especially in HD negative cases, is crucial to limit recurrence.

Colonic volvulus represents a pediatric condition (mean reported age of onset is 7–12 years), determining about 3–5% of all intestinal obstructions [13,21]. However, an underestimated incidence cannot be rejected because most cases remain unreported.

We observed a slightly higher mean age at diagnosis among females with respect to males, consistent with the findings of Colinet et al. [22].

CV usually involves the sigmoid, followed by the cecum, while transverse colon and splenic flexure volvuli are extremely uncommon (3% of all CV) [6].

The exact etiopathogenesis remains unknown. Presumed anatomical, mechanical and physiological risk factors include: previous abdominal surgery, omphalomesenteric abnormalities, excessive colonic mobility, long colonic mesentery, malrotation, psychiatric disorders, bowel distension secondary to chronic constipation or distal obstruction due to anal stenosis, developmental delay and HD [8,13,21,23,24]. Neurodevelopmental delay, associated with constipation, longer transit time and colonic dysmotility, was the most common underlying cause in the series by Marine et al. and Folaranmi et al. [25,26]. This finding may be attributable to the fact that patients with neurological impairment may be on ketogenic diets with lower intake of water and fiber, with often concurrent use of anticonvulsants, opioids and antispasmodic medications. HD may be a risk factor for CV and requires biopsy screening [18,27]. In general, it seems that a chronically distended colon leads to elongation of the mesentery and to an altered colonic motility. This anatomical configuration favors the approximation of the two limbs of the sigmoid colon and the torsion of the sigmoid colon around the mesenteric axis [28].

In accordance with the literature, most of our patients reported chronic constipation. HD was diagnosed in 27.3% of the cases, a higher incidence than that recently reported by Atamanalp (0.2% of sigmoid volvulus cases) [16], but we should consider that the author practices in an area where CV is endemic (eastern Anatolia) and may show some differences from our series. Moreover, volvulus is a rare complication of HD, but the reported incidence of HD among children with volvulus is 18% [29]. That being said, we also had 18% of patients with elongated mesentery and no chronic history of constipation. We should also consider the possible involvement of the brain–gut axis (GBA) in the pathophysiological mechanism of CV, as we observed associated neurodevelopmental diseases in more than half of cases (54.4%). The link between dysbiosis and central nervous system disorders has been proven in clinical practice [30,31]. The complexity of GBA interactions encompasses immune overactivation, intestinal permeability, enteric reflexes and entero-endocrine signaling. GBA influences and modulates peripheral intestinal functions [30,31].

From the clinical point of view, CV is a potential surgical emergency determined by bowel strangulation with perforation, peritonitis and sepsis, requiring surgery and resulting in high morbidity and mortality. Vascular impairment and severe bowel obstruction are more likely to happen with torsions greater than 180 degrees [13]. There exists also a second possible clinical pattern: a gradual and chronic form presenting with recurrent episodes of abdominal distension and pain, often self-limiting and related to sigmoid CV [13,24,28,32]. In the latter case, treatment by contrast enema and/or endoscopy has been effectively employed, followed by elective surgical resection [13,22,28]. Surgery is advocated as the treatment of choice to avoid recurrences, which have reported rates of up to 35% [7,19,22,32,33]. Surgical options include resection of the dilated bowel or colopexy, the latter being indicated in high-risk patients. Simple surgical detorsion should be discouraged because the recurrence rate is similar to that after sigmoidoscopy (32%) [7,19,22]. In our experience, all patients presented with signs of acute bowel obstruction. There were seemingly no diagnostic delays. However, we may suppose that patients with developmental delay or younger age may not communicate as well as other children and the seeking of medical help may occur later. Radiological and endoscopic detorsions have been successful in 71.4% of sigmoid CV. Data on non-surgical management are non-univocal: some authors reported 30–40% success with non-surgical reduction of sigmoid volvulus but others claim 100% success [7,34]. On the other hand, they agree that volvulus involving other colonic segments more often needs surgery [7,34]. In the one patient with sigmoidoscopy failure, the patient’s diagnosed HD may have played a causative role in that failure, but this remains purely speculation. Sigmoid resection was a reliable approach, and minimally invasive surgery was demonstrated to be safe and effective in 80% of patients. The presence of severe dilatation may interfere with laparoscopy, as we experienced in one patient. The planning of the definitive surgery 1–2 months after volvulus detorsion, with proper preparation (dietary modifications, daily enemas and fecal softeners) to treat constipation, may obviate this problem. A more aggressive surgical approach was required when the CV involved the transverse colon [6].

At follow-up, we observed that patients who underwent total colectomy developed proximal intestinal dilatation. Indeed, specific aspects of patients with colonic volvulus are yet to be understood.

We feel that this aspect requires more in-depth analysis. Data in the literature on patients’ follow-up focus on short-term results, such as recurrences in patients treated conservatively [22,24,35]. Moreover, most of the articles do not report results of the screening for associated anomalies, and they do not explicitly say if they carried out screening.

**Figure 3:**
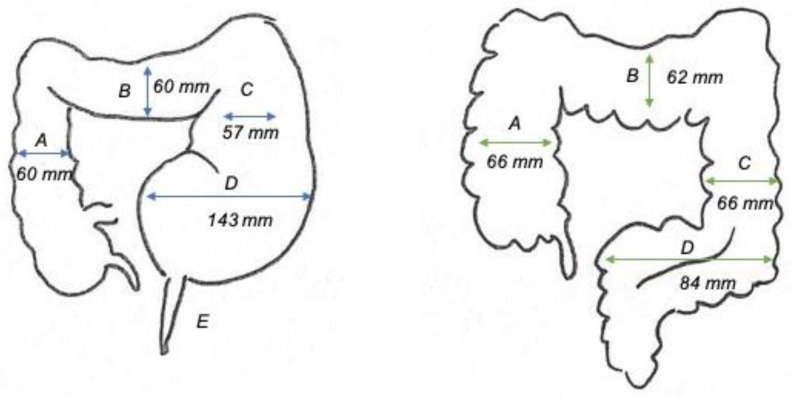
Schematic representation of the colonic frame in Hirschsprung’s disease (HD) patients and non-HD patients with colonic volvulus (E = narrow aganglionic colon, D = sigmoid, C = descending colon, B = transverse colon, A = ascending colon).

In our series, radiological studies showed significant sigmoid dilatation. Patients with HD and volvulus seem to have greater sigmoid diameters (mean diameter 143 vs. 84 mm of non-HD volvulus), but the descending, transverse and ascending colon showed reduced caliber (mean 59 mm). Intestinal diameters in cases with non-HD volvulus were closer to the physiological dimensions of the colon (mean 65 mm), as indicated in Figure 3. Late-diagnosed short-segment HD increases the likelihood of developing CV because it determines chronic and prolonged constipation [21,23]. Rectal biopsy is indicted in patients with CV to rule out HD or other forms of hypoganglionosis [7]. Definitive surgery is then planned according to the results of the rectal biopsy and, in our experience, this was associated with no CV recurrences.

According to Atamanalp, the numerical density of ganglion cells correlates to volvulus onset, and it is feasible that HD may trigger the sigmoid twisting [16]. Fujiya et al. confirmed that a decrease in the quantity of enteric plexus and ganglion cells precedes the clinical manifestation of the volvulus [36].

Our radiological findings may be interpreted as arguments supporting the GBA hypothesis, propelling us to take greater care in evaluating these patients, especially those without HD. Special attention should also be paid to the follow-up since, after surgical removal of the ileo-cecal valve in one patient, there was the development of a massive dilatation of the previously normal proximal small intestine, suggesting that other elements may play a role in the “gastrointestinal homeostasis” of these patients.

The findings of this study have to be seen in the light of some limitations. The first is the small number of patients, which limits the quantitative analysis. The second concerns the scarce literature concerning this topic, with many papers being based on single case reports, limiting the possibility to perform significant analysis and to draw definitive conclusions. Further studies on larger series are necessary to confirm our results, and a meta-analysis may be proposed to fill the gap and better understand this condition.

## 5. Conclusions

Colonic volvulus is a challenging condition that requires prompt patient care. Surgery is indicated to avoid recurrences and to treat complications but should be performed after careful evaluation of the patient and the exclusion of associated anomalies, including HD, alpha-actin deficiency, neurological, metabolic and immunologic diseases and CIPO. Preoperative management should include genetic and endocrinological counseling together with pediatric and neurologic evaluation. Extensive resections should be reserved to select severe cases, often involving the transverse colon. Many pathophysiologic aspects remain unclear, and lifelong follow-up is therefore necessary for monitoring of the proximal gastrointestinal tract. 

## Figures and Tables

**Table 1:** Patients’ characteristics.

Pt	Sex	Age (Years)	Associated Anomalies	Previous Abdominal Surgery	History of Chronic Constipation	Segment Involved
1	M	15	None	None	No	Sigmoid
2	F	15	Hirschsprung’s disease	None	Yes	Sigmoid
3	M	15	None	None	No	Sigmoid
4	F	10	Hirschsprung’s disease	None	Yes	Sigmoid
5	M	7	None	None	Yes	Sigmoid
6	F	14	Cerebral palsy, autism, anterior anus; simultaneous splenic, gastric and pancreatic volvulus	None	Yes	Sigmoid
7	F	11	None	None	Yes	Sigmoid
8	F	15	Acquired cerebral palsy	None	Yes	Sigmoid
9	F	15	Hirschsprung’s disease; Ito syndrome, cerebral palsy	None	Yes	Transverse
10	M	11	Infantile cerebral palsy post meningoencephalitis	Yes (ventriculoperitoneal shunt placement)	Yes	Transverse
11	F	14	Dystonic spastic tetraparesis, kernicterus	Yes (videolaparoscopic surgery, Nissen, gastrostomy)	Yes	Total colon

**Table 2:** Mean colon diameter based on bowel enema evaluation; HD = Hirschsprung’s disease.

	Sigmoid Diameter (mm)	Left Colon Diameter (mm)	Transverse Diameter (mm)	Right Colon Diameter (mm)
All cases	102 ± 41	63 ± 18	61 ± 17	64 ± 17
HD patients	143 ± 49	57 ± 17	60 ± 20	60 ± 23
Non-HD patients	84 ± 22	66 ± 19	62 ± 18	66 ± 16

**Table 3:** Management of patients and outcome.

Pt	Associated Anomalies	Treatment Upon Admittance (Effect)	Surgery	Follow-Up
1	None	Endoscopic detorsion (success)	Sigmoid resection (VLS converted to open)	Uneventful
2	Hirschsprung’s disease	Endoscopic detorsion (failure: sigmoid resection and colostomy)	Soave pull-through (open)	Uneventful
3	None	Endoscopic detorsion (recurrency)	Left hemicolectomy (VLS-SILS port)	Uneventful
4	Hirschsprung’s disease	Endoscopic detorsion (success)	Soave pull-through (VLS)	Uneventful
5	None	Radiologic detorsion (success)	Sigmoid resection (VLS)	Uneventful
6	Psychomotor retardation, autism, anterior anus; simultaneous splenic, gastric and pancreatic volvulus	Surgery: sigmoid detorsion, splenectomy, temporary ileostomy (success)	Total colectomy (VLS)	Anastomotic stenosis (redo anastomosis); periodic episodes of intestinal sub-obstruction
7	None	Radiologic detorsion (success)	Sigmoid resection (VLS)	Uneventful
8	Acquired psychomotor retardation	Radiologic detorsion (success)	Refused by parents	N/D
9	Hirschsprung’s disease; Ito syndrome, psychomotor retardation	Colostomy (persistent intestinal obstruction)	Total colectomy, pouch ileoanal anastomosis, ileostomy (open)	Uneventful
10	Infantile cerebral palsy post meningoencephalitis	Surgery: transverse detorsion and colopexy (persistent intestinal obstruction)	Total colectomy, ileostomy (open)	Two episodes of intestinal sub-obstruction
11	Dystonic spastic tetraparesis, kernicterus	Surgery: detorsion and colopexy (open)	No	Uneventful

VLS = videolaparoscopic surgery, SILS= single incision laparoscopis surgery; N/D = not defined.

## Data Availability

Data reported in this study are available upon request from the corresponding author.
